# *Encephalitozoon cuniculi* Microsporidia in Cerebrospinal Fluid from Immunocompetent Patients, Czech Republic

**DOI:** 10.3201/eid3006.231585

**Published:** 2024-06

**Authors:** Bohumil Sak, Katka Mansfeldová, Klára Brdíčková, Petra Gottliebová, Elka Nyčová, Nikola Holubová, Jana Fenclová, Marta Kicia, Żaneta Zajączkowska, Martin Kváč

**Affiliations:** Biology Centre of the Czech Academy of Sciences, České Budějovice, Czech Republic (B. Sak, N. Holubová, J. Fenclová, M. Kváč);; University of South Bohemia, České Budějovice (K. Mansfeldová, J. Fenclová, M. Kváč);; Bulovka Hospital, Prague, Czech Republic (K. Brdíčková, P. Gottliebová, E. Nyčová);; Wroclaw Medical University, Wroclaw, Poland (M. Kicia, Ż. Zajączkowska)

**Keywords:** Encephalitozoon cuniculi, parasites, zoonoses, cerebrospinal fluid, PCR, qPCR, latent infection, Czech Republic

## Abstract

We retrospectively analyzed of 211 frozen cerebrospinal fluid samples from immunocompetent persons in the Czech Republic and detected 6 *Encephalitozoon cuniculi*–positive samples. Microsporidiosis is generally underestimated and patients are not usually tested for microsporidia, but latent infection in immunodeficient and immunocompetent patients can cause serious complications if not detected and treated.

Microsporidia are obligate intracellular parasites of invertebrate and vertebrate hosts and are considered to be a sister group to fungi (*1*). Of the 1,300 species in >200 genera that have been described (*2*), *Encephalitozoon cuniculi*, especially genotypes I and II, is the most common in humans (*3*,*4*).

Although the digestive tract represents a port of entry, *Encephalitozoon* infections can occur in almost every organ system and can cause various diseases (*4*). Encephalitozoonosis is a serious problem in immunodeficient hosts, including HIV-positive patients and patients on immunosuppressive treatments. In immunocompetent persons, microsporidial infections are predominantly chronic and asymptomatic (*5*).

Recent studies have described engagement of macrophages, or other immune cells involved in the development of inflammation, serving as vehicles and transporting microsporidia toward target tissues outside the intestines (*6*,*7*). Microsporidia are often overlooked in clinical samples because diagnosis is problematic, but hidden infections can cause tremendous multisystem damage and various nonspecific pathologies, and few effective treatments are available (*8*). We evaluated the incidence of generally neglected *Encephalitozoon* spp. in immunocompetent patients by retrospectively analyzing previously collected cerebrospinal fluid (CSF) samples. 

## The Study

Bulovka Hospital, Prague, Czech Republic, provided 211 CSF samples that had been deep frozen at −80°C. CSF samples were collected from immunocompetent patients; the only other patient data reported were the year of birth and sex. We obtained total DNA from sediments obtained from thawed CSF together with extraction negative control in each series, as previously described (*6*). We used the same methods to isolate control DNA from purified *E. intestinalis* spores. The study was conducted beyond the routine screening of existing unused specimens and focused on potential detection of microsporidia in CSF recovered from immunocompetent patients hospitalized at 1 hospital. Because the study was performed using anonymized samples with no intervention tract, patient consent was not required.

We used an *Encephalitozoon* spp.*–*specific nested PCR to amplify the internal transcribed spacer region (*9*,*10*). We included DNA of *E. intestinalis* microsporidia as a PCR-positive control and ultrapure water as a negative control and evaluated PCR products by gel electrophoresis.

We quantified DNA from PCR-positive samples by using reverse transcription PCR to amplify a 268-bp region of the 16S rRNA gene of *E. cuniculi* (*10*). Each run included unspiked specimens and diluent blanks. We considered results positive when the fluorescence signal crossed the baseline at <43 cycles. We used a standard curve to calculate the total number of spores in 1 mL of each sample.

 We used the QIAquick Gel Extraction Kit (QIAGEN, https://www.qiagen.com) to purify PCR amplicons of the internal transcribed spacer region and submitted amplicons to SEQme (https://www.seqme.eu) for sequencing in both directions. We manually edited nucleotide sequences by using the ChromasPro 2.1.4 program (Technelysium, https://technelysium.com.au) and used MAFFT version 7 (http://mafft.cbrc.jp) to align sequences with reference GenBank submissions. We also microscopically examined PCR-positive samples. We air dried a drop of CSF, fixed it with methanol, and stained with standard Calcofluor M2R (Sigma-Aldrich, https://www.sigmaaldrich.com) (*11*).

Of 211 CSF samples examined, 115 were from male patients and 96 from female patients. The median patient age was 34.0 (range 2–81) years (Table). Among all samples, 6 were positive for microsporidia DNA, 0.9% (1/115) of samples from male and 5.2% (5/96) of samples from female patients. The age of positive patients ranged from 13 to 75 years (median 45.5 years). The spore concentration in samples was 30–500 spores/mL. 

**Table:** Characteristics of patients in a study of *Encephalitozoon cuniculi* in cerebrospinal fluid from immunocompetent patients, Czech Republic

Sex	Total no. sampled	Median age (range)	*E. cuniculi­*–positive patients			Sample testing results	
			Patient no.	Age, y		Nested PCR genotype	RT-PCR quantification/mL (Ct)
M	115	34.0 (4–81)	56	63		*E. cuniculi* II	3.0 × 10^1^ (39)
F	96	33.5 (2–80)	54	13		*E. cuniculi* II	5.7 × 10^1^ (38)
			139	45		*E. cuniculi* II	1.1 × 10^2^ (36)
			185	48		*E. cuniculi* II	5.1 × 10^2^ (35)
			194	75		*E. cuniculi* II	3.0 × 10^1^ (38)
			197	32		*E. cuniculi* II	1.0 × 10^1^ (39)
Total	211	34.0 (2–81)					

**Figure 1:**
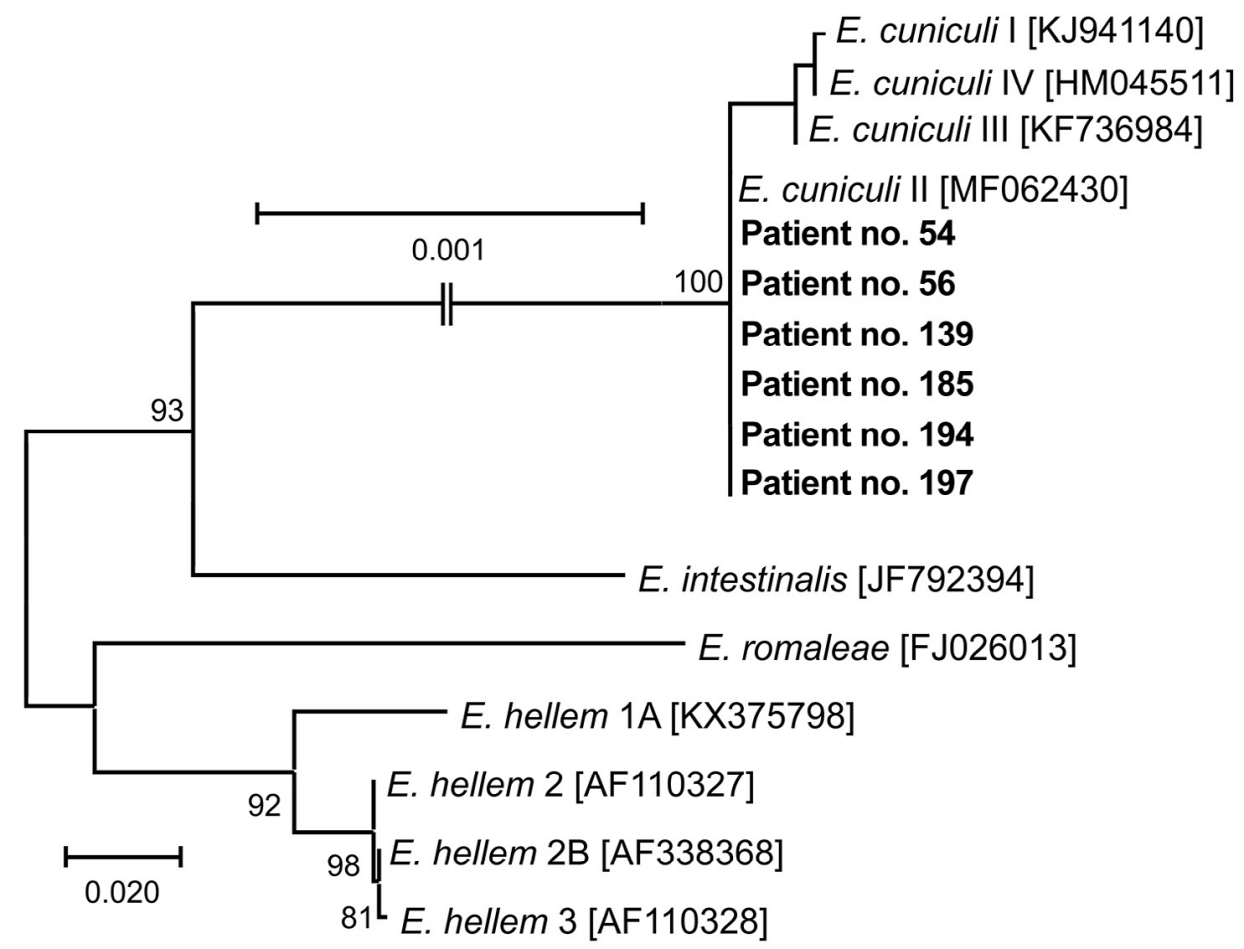
Phylogenetic analysis of *Encephalitozoon cuniculi* genotypes recovered from cerebrospinal fluid of immunocompetent patients, Czech Republic. Bold indicates sequences obtained in this study, identified by patient number. Sequences for comparisons were obtained from GenBank; accession numbers are in brackets. Tree was constructed by using the maximum-likelihood method. Partial sequences of 16S rRNA gene, the entire internal transcribed spacer region, and a partial sequence of 5.8S rRNA gene were inferred by using neighbor-joining analyses, and relationships were computed by using the Tamura 3-parameter method with gamma distribution and parametric bootstrap analysis of 1,000 replicates in MEGA X software (MEGA, https://www.megasoftware.net). Scale bar indicates nucleotide substitutions per site.

**Figure 2:**
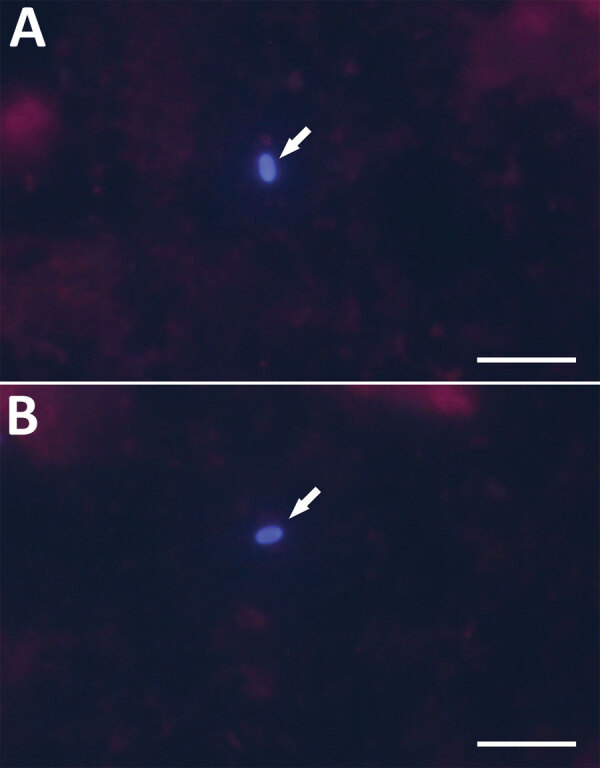
Microscopic examination *Encephalitozoon cuniculi*­–positive cerebrospinal fluid from immunocompetent patients, Czech Republic. Microsporidial spores (arrows) were stained with Calcofluor M2R and viewed in 490 nm. A) Patient no. 139; B) patient no. 185. Scale bar indicates 10 µm.

Sequence analyses revealed 100% identity to *E. cuniculi* genotype II (GenBank accession no. MF062430) in all positive samples (Table; Figure 1). Microscopic analysis of Calcofluor M2R–stained smears confirmed the presence of spores (1–2 spores per slide) in samples obtained from 2 patients, nos. 139 and 185, who had the highest *Encephalitozoon* DNA burden (Figure 2). The other 4 patients were microscopically negative.

## Conclusions

Although microsporidiosis is mainly detected in immunodeficient patients, data from the literature imply that otherwise healthy persons also are at risk (*12*,*13*). Those data indicate that apparently healthy persons could be infected without any clinical signs, and the risk increases with age (*12*). Whether microsporidial infection potentially leads to a deterioration in health that could be life-threatening in the event of a decline in immunity has not been determined (*12*,*14*,*15*).

The fecal–oral route is generally accepted as the most common transmission route because the spores are passed in the urine or feces of infected patients, then mostly contaminate water sources. Moreover, possible foodborne transmission, including through fresh vegetables and fruits, milk, cheese, and fermented meat products, has been reported (*13*). Besides those transmission routes, respiratory tract infection suggests airborne transmission by contaminated aerosols (*13*).

Microsporidia are small intracellular fungi capable of causing widespread infections within a few days, despite their lack of active motility and limited spreading possibilities (*14*). The exact spreading mechanism is still unknown; however, the possible connection between activation of proinflammatory cellular immune response and targeted transport of microsporidia toward inflammation site has been proposed on the basis of clinical and experimental data (*6*,*7*,*11*).

In this study, we detected microsporidia DNA in 3% of tested CSF samples from 211 patients of one hospital. The molecular data were supported by microscopy in 2 patients who had the highest spore loads. Although the other 4 PCR-positive patients tested microscopically negative, those results could be caused by limited sensitivity of microscopy in low burden samples, rather than laboratory contamination. Because we obtained uniform results from specific patients using both PCR and quantitative PCR, contamination is unlikely. Moreover, we can exclude laboratory contamination because the same trained personnel took the samples and ran PCRs under sterile conditions. In addition, PCR diagnostic laboratory is structurally divided into separate areas that adhere to the 1-direction workflow, and all negative controls used in sample processing were negative.

Our results for microsporidia detection indicate an increasing prevalence of latent microsporidiosis with patient age, which is consistent with the results of previous studies (*12*). Moreover, the presence of microsporidia in CSF represents a potentially serious condition; unfortunately, we cannot infer any association with the clinical condition of the patients because we did not have patient histories or reasons for collecting CSF samples. However, we can assume a possible link between the patients’ health issues and the presence of microsporidia in CSF, similar to those found in another study (*15*). That study reported a case of a paralyzed patient with a right frontal lobe abscess containing *E. cuniculi* genotype I; the patient was successfully treated following appropriate treatment regimen.

In conclusion, disseminated latent microsporidiosis can cause several serious diseases with nonspecific symptoms and ambiguous etiology that can be life-threating or fatal if misdiagnosed and left untreated. We encourage increased awareness of latent microsporidiosis and development of targeted screening that enables timely treatment.

## References

[1] Keeling PJ, Doolittle WF. Alpha-tubulin from early-diverging eukaryotic lineages and the evolution of the tubulin family. Mol Biol Evol. 1996;13:1297–305. 10.1093/oxfordjournals.molbev.a025576 8952074

[2] Cali A, Becnel JJ, Takvorian PM. Microsporidia. In: Archibald JM, Simpson AGB, Slamovits CH, editors. Handbook of the protists. Cham (Switzerland): Springer; 2017. p. 1559–618.

[3] Didier ES. Microsporidiosis: an emerging and opportunistic infection in humans and animals. Acta Trop. 2005;94:61–76. 10.1016/j.actatropica.2005.01.010 15777637

[4] Didier ES, Weiss LM. Microsporidiosis: not just in AIDS patients. Curr Opin Infect Dis. 2011;24:490–5. 10.1097/QCO.0b013e32834aa152 21844802 PMC3416021

[5] Weber R, Bryan RT, Schwartz DA, Owen RL. Human microsporidial infections. Clin Microbiol Rev. 1994;7:426–61. 10.1128/CMR.7.4.426 7834600 PMC358336

[6] Brdíčková K, Sak B, Holubová N, Květoňová D, Hlásková L, Kicia M, et al. *Encephalitozoon cuniculi* genotype II concentrates in inflammation foci. J Inflamm Res. 2020;13:583–93. 10.2147/JIR.S271628 33061524 PMC7524191

[7] Sak B, Holubová N, Květoňová D, Hlásková L, Tinavská J, Kicia M, et al. Comparison of the concentration of *Encephalitozoon cuniculi* genotypes I and III in inflammatory foci under experimental conditions. J Inflamm Res. 2022;15:2721–30. 10.2147/JIR.S363509 35502243 PMC9056047

[8] Lallo MA, da Costa LF, de Castro JM. Effect of three drugs against *Encephalitozoon cuniculi* infection in immunosuppressed mice. Antimicrob Agents Chemother. 2013;57:3067–71. 10.1128/AAC.00157-13 23612191 PMC3697356

[9] Katzwinkel-Wladarsch S, Lieb M, Helse W, Löscher T, Rinder H. Direct amplification and species determination of microsporidian DNA from stool specimens. Trop Med Int Health. 1996;1:373–8. 10.1046/j.1365-3156.1996.d01-51.x 8673842

[10] Wolk DM, Schneider SK, Wengenack NL, Sloan LM, Rosenblatt JE. Real-time PCR method for detection of *Encephalitozoon intestinalis* from stool specimens. J Clin Microbiol. 2002;40:3922–8. 10.1128/JCM.40.11.3922-3928.2002 12409353 PMC139654

[11] Kicia M, Wesolowska M, Kopacz Z, Kvác M, Sak B, Sokulska M, et al. Disseminated infection of *Encephalitozoon cuniculi* associated with osteolysis of hip periprosthetic tissue. Clin Infect Dis. 2018;67:1228–34. 10.1093/cid/ciy256 29659738

[12] Sak B, Kváč M, Kučerová Z, Květoňová D, Saková K. Latent microsporidial infection in immunocompetent individuals - a longitudinal study. PLoS Negl Trop Dis. 2011;5:e1162. 10.1371/journal.pntd.0001162 21629721 PMC3101169

[13] Sak B, Kváč M. Chronic infections in mammals due to microsporidia. In: Weiss LM, Reinke AW, editors. Microsporidia: current advances in biology. Cham (Switzerland): Springer Experientia Supplementum; 2022. p. 319–71. 10.1007/978-3-030-93306-7_12 35544008

[14] Kotková M, Sak B, Květoňová D, Kváč M. Latent microsporidiosis caused by *Encephalitozoon cuniculi* in immunocompetent hosts: a murine model demonstrating the ineffectiveness of the immune system and treatment with albendazole. PLoS One. 2013;8:e60941. 10.1371/journal.pone.0060941 23593356 PMC3623998

[15] Ditrich O, Chrdle A, Sak B, Chmelík V, Kubále J, Dyková I, et al. *Encephalitozoon cuniculi* genotype I as a causative agent of brain abscess in an immunocompetent patient. J Clin Microbiol. 2011;49:2769–71. 10.1128/JCM.00620-11 21593268 PMC3147860

